# Ki-67 protein expression and tumor associated inflammatory cells (macrophages and mast cells) in canine colorectal carcinoma

**DOI:** 10.1186/s12917-017-1030-7

**Published:** 2017-04-20

**Authors:** M. Woldemeskel, I. Hawkins, L. Whittington

**Affiliations:** Department of Pathology, Tifton Veterinary Diagnostic and Investigational Laboratory, College of Veterinary Medicine, University of Georgia, 43 Brighton Rd, Tifton, GA 31793 USA

**Keywords:** Canine, Colorectal carcinoma, Ki67, Macrophage, Mast cell

## Abstract

**Background:**

Ki67 index, tumor associated macrophages (TAMs) and mast cells (MCs) are associated with malignancies in animal and human neoplasms including colorectal carcinomas (CRC). This has not been assessed in canine CRC. Given similar genetic abnormalities between human and canine CRC, we assessed Ki-67 and mitotic indices, TAMs and MC count (MCC) in canine CRC (*n* = 17). TAMs and MCC were compared with those in adenomas (*n* = 13) and control (*n* = 9).

**Results:**

Ki-67 index in CRC (17.13 ± 11.50) was strongly correlated (*r* = 0.98, *p* < 0.05) with mitotic index (3.52 ± 1.80). MCC was higher (*p* < 0.05) in CRC (6.30 ± 3.98) than in adenomas (0.78 ± 0.77) and control (0.35 ± 0.33). The results suggest that Ki-67 index and MCC are associated with malignancy in canine CRC. Higher average TAMs were counted in adenomas (21.30 ± 20.70) and in CRC (11.00 ± 9.82) than in the control (7.69 ± 7.26), although the differences were not significant (*p* > 0.05).

**Conclusion:**

Ki-67 index, TAMs and MCC in canine CRC were recorded for the first time in this study. Ki-67 index and MCC are associated with malignancy in canine CRC. Quantitative assessment of MCs and Ki-67 coupled with mitotic index and other clinical parameters may help in evaluating malignancy in canine CRC. TAMs likely play a role in the development of canine colorectal tumors. Further studies to determine the clinical significance of these parameters for prognostic, chemo-preventive and chemotherapeutic purposes in canine colorectal tumors are recommended.

## Background

Colorectal cancer (CRC) is one of the most frequent and most serious malignancies in humans [1–3]. It is one of the most common malignant cancers and among the leading causes of cancer-related deaths worldwide [1, 4, 5]. Other than humans, the only domestic species that naturally develops intestinal cancer with any frequency is the dog, which shares similar environment and in some households similar diet with humans [6]. Colorectal tumors are among the most common gastrointestinal neoplasms in the dog. Up to 60% of all canine intestinal tumors are located in the colorectum, where adenocarcinomas are relatively common [7, 8].

Ki-67 is widely applied in routine clinical work [9] and has been studied in relation to the development and progression of human CRC [2]. It was considered as an important predictive parameter in human CRC. Lumachi et al. [10] reported that there is an inverse correlation between overall survival and the percentage of Ki-67-positive tumor cells and that Ki-67 overexpression in CRC is associated with a worse outcome. However, some studies have failed to demonstrate its prognostic significance [11]. While some reported association of increased Ki-67 expression with poor prognosis [12], others [13] reported a good prognosis associated with Ki-67 expression. Although its prognostic value remains controversial [14], recently, Melling et al. [15] reported higher Ki-67 expression as an independent prognostic marker in human CRC.

Development of CRC has been associated with chronic inflammation of the large bowel elicited by various causes. Macrophages are among the inflammatory cells most involved in these processes [16, 17]. Pro-inflammatory cytokines released by macrophages are considered as major agents in the transition between inflammation and inflammation-related CRC [18]. Mast cells (MCs) are also among inflammatory cells involved in tumor development and progression in various human [19, 20] and animal [21–23] neoplasms. MCs can exert pro-tumor effects by influencing the microenvironment or, directly, by conditioning the fate of tumor cells including drug resistance [24]. Adenomatous polyps from which CRC develops are characterized by high mast cell count (MCC) [25] and existing polyps showed significant remission upon MC depletion in experimental mouse model [26]. It was also demonstrated that presence of MCs, particularly their ability to degranulate, is indispensable for tumor progression in a Myc- driven model of pancreatic cancer [27].

Genetic and molecular similarities as well as similar molecular and genetic pathways of cancer development and progression between human and dog CRCs have been suggested [28]. Ki-67 expression in CRC, and tumor associated macrophages and mast cells within colorectal tumors in dogs were not previously reported. We hypothesize that high Ki-67 expression and tumor associated macrophages and mast cells correlate with malignancy in canine CRC. Given genetic similarities of human and canine CRC, the objective of this study was to determine the Ki-67 index in canine CRC and assess its correlation with tumor malignancy, tumor associated macrophages (TAMs) and MCs. TAMs and MCs in canine CRC were evaluated and compared with those in colorectal adenomas and non-tumorous colorectal tissues.

## Methods

Surgical biopsy specimens from colorectal tissues of 39 dogs of various breeds with carcinoma (*n* = 17), adenoma (*n* = 13) and non-neoplastic colorectal tissues from dogs with no colorectal tumors as control (*n* = 9) archived at the Tifton Veterinary Diagnostic and Investigational Laboratory, College of Veterinary Medicine, The University of Georgia, USA, were used in the study. The tissues were fixed in 10% buffered formalin solution, processed for routine histology, paraffin embedded, sectioned at 4–5 μm, stained with standard Hematoxylin-Eosin and Toluidine blue (Sigma, St. Louis, USA) stains and examined by light microscopy. The specimens were evaluated by two board certified pathologists. Mitotic cells were counted in 10 high power fields (HPFs/40× objectives) and mitotic index was calculated by dividing the counts (results) by 10. Tumor malignancy was assessed based on tumor size at time of biopsy, mitotic index, necrosis and vascular invasion. MCC was made on Toluidine blue (Sigma, St. Louis, USA) stained sections. Ki-67 expression and tumor associated macrophage (TAM) count were assessed using immunohistochemistry against Ki-67 protein and macrophage marker. The average MCC and TAMs was made by counting the cells in 10 high power fields (40×).

### Immunohistochemistry (IHC)

The tissue sections were subjected to IHC using antibodies against Ki-67 protein and macrophage/histiocyte marker. Immunohistochemical staining procedures for Ki-67 (Mouse-anti-KI-67, Clone 7B11; Zymed® Laboratories; Invitrogen immunodetection) and macrophage marker (Myeloid/histiocytes antigen; monoclonal-mouse, Clone MAC 387, DAKO Carpinteria, CA) were applied briefly as follows. Tissue samples were cut at 4–5 μm sections, deparaffinized by xylene-ethanol sequence, and rehydrated in graded ethanol solutions. Antigen retrieval for Ki-67 IHC was made by Heat Induced Epitope Retrieval (HIER) using a 1:10 diluted Target Retrieval Solution, pH 6 (DAKO). Heat was supplied by a Black and Decker Vegetable steamer for 25 min, followed by 10 min in the hot solution on the counter top. Endogenous peroxidase was blocked using UltraVision Hydrogen Peroxide Block for 10 min. Non-specific background staining was blocked by incubating the samples for 5 min using UltraVision Protein Block (Thermo Scientific; Lab Vision Corp, Ca, USA). Each step of incubation was followed by a thorough washing of the sections with Tris Buffered Saline (TBS) at pH 7.4. The sections were incubated for 1 h at room temperature with primary antibodies against Ki-67 and macrophage marker (Clone MAC 387) at 1:25 and 1:200 dilutions, respectively. The sections were then incubated for 10 min with a Primary Antibody Amplifier Quanto and later followed by incubation with HRP Polymer Quanto (Thermo Scientific; Lab Vision Corp, Ca, USA) for 10 min. The sections were rinsed with TBS after each step and were later incubated with DAB Quanto Chromagen and DAB Quanto Substrate mixture (DAB, Quanto) for 5 min. Finally, all sections were counterstained with Gill’s Hematoxylin (Gills III- Formula, Surgipath Richmond, IL, USA). Negative control sections were made by substituting TBS for the primary antibodies.

### Ki-67 expression

According to the International Ki-67 Breast Cancer Working Group [29], the Ki-67 evaluation method can be based on three patterns of Ki-67 immunostaining: homogeneous, hot spots, and a gradient of increasing staining toward the tumor edge. The most commonly used method to calculate the Ki-67 index is based on the hot spots [30]. The choice of the hot spots counting is generally based on the assumption that the areas with a high proliferation index potentially predict a more aggressive biological behavior of the neoplasm [31]. In this study, the hot-spot based counting was employed. The labeled sections were screened at 10× (lower) magnification to detect areas with distinct staining (hot-spots). In each section, the most intense three areas stained with Ki-67 were identified and at least 500 cells were counted in each area at a high power field of the microscope (40×) and also using computer assisted Image-J cell counting method. Areas with severe inflammation and necrosis were avoided. A ratio of the number of nuclei stained positively with Ki-67 antigen was determined in each area and the average of these ratios was considered as Ki-67 index as previously described [31].

### Statistical analysis

The Ki-67 labelling index (%) was calculated by dividing the total Ki-67 positive cells by the total numbers of cells multiplied by 100. Mitotic index was determined by dividing the number of mitotic cells in 10 HPFs by 10. MCC and TAMs count were made in 10 high power fields (40×) of the microscope and the results were given as mean ± standard deviation (SD). Data were analyzed using analysis of variance. Correlation between the various parameters was determined using Spearman’s correlation coefficient. In all cases, the results were considered statistically significant if *p* values were <0.05.

## Results

A variety of breeds were included in the study. These included mixed breeds (*n* = 6), Labrador retriever (*n* = 4), Basset Hound (*n* = 2), Cocker Spaniel (*n* = 2), pointer (*n* = 2), unknown breed (*n* = 6) and a variety of other breeds represented only once. The average age of the dogs was 7.5 years (range was 7–12 for carcinomas, 2–11 for adenomas and 1–9 for the control). There was no statistically significant age difference between the groups.

**Fig. 1:**
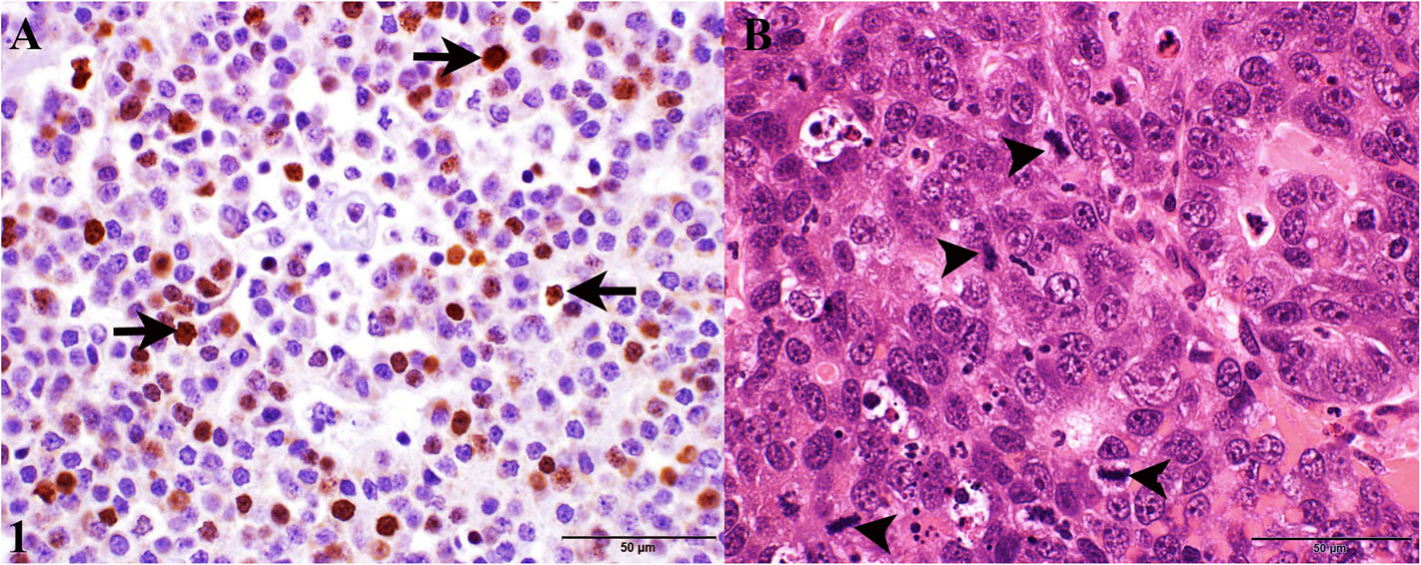
Canine colorectal carcinoma. **a** Immunoreactivity of tumor cell nuclei for Ki-67 protein (arrows). Bar = 50 μm. **b** Hematoxylin-eosin stained section showing mitotic cells (arrow heads). Hematoxylin-eosin. Bar =50 μm

The Ki-67 and mitotic indices (mean ± SD) in carcinomas were 17.13 ± 11.50 and 3.52 ± 1.80, respectively. A strong positive correlation (*r* = 0.98, *p* < 0.05) was recorded between the Ki-67 and mitotic indices. The Ki-67 expression was variable and in some diffuse expression (Fig. 1) was present while in other cases in which only a few Ki-67 positive cells were present, the expression was observed only in mitotic cells. Ki-67 was associated (*r* = 0.71; *P* < 0.05) with tumor malignancy based on tumor size at times of biopsy, mitotic index, necrosis and vascular invasion.

**Fig. 2:**
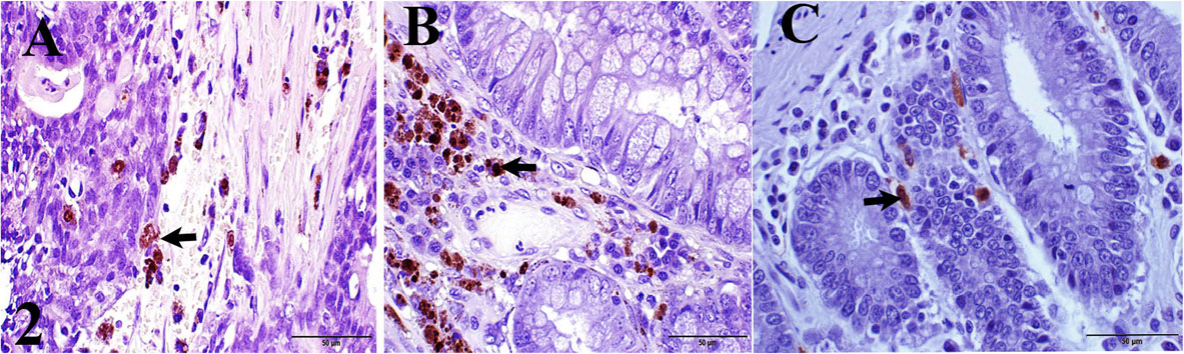
Canine colorectal tissues from: **a** Carcinoma **b** Adenoma and **c** Non-neoplastic tissue (control), showing TAMs positive immunoreactivity (arrows) against macrophage marker (MAC 387). Bar = 50 μm

Higher average TAMs were observed in adenomas (21.30 ± 20.70) and in CRC (11.00 ± 9.82) than in non-neoplastic tissues (7.69 ± 7.26) (Fig. 2 and Table 1). The differences, however, were not statistically significant (*p* > 0.05). Although not significant (*p* > 0.05), TAMs in CRC were also, positively, but weakly correlated with mitotic (*r* = 0.35) and Ki-67 (*r* = 0.25) indices, and MCC (*r* = 0.24).

**Table 1:** Average MCC ± SD and TAMs ± SD in canine colorectal tissues

	Carcinoma (*n* = 17)	Adenoma (*n* = 13)	Control (*n* = 9)	*P*-value
TAMs	11.00 ± 9.82^a^	21.30 ± 20.70^a^	7.69 ± 7.26^a^	>0.05
MCC	6.30 ± 3.98^b^	0.78 ± 0.77^a^	0.35 ± 0.33^a^	<0.05

Different superscript letters in the same row indicate statistically significant differences (*p* < 0.05). *MCC* mast cell count, *TAMs* tumor associated macrophages, *SD* standard deviation

**Fig. 3:**
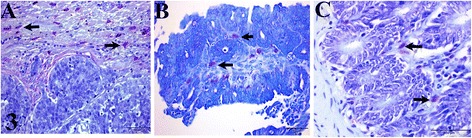
Canine colorectal tissues from: **a** Carcinoma **b** Adenoma and **c** Non-neoplastic tissue (control), showing Toluidine blue stained mast cells (arrows). Toluidine blue stain. Bar = 50 μm

The average MCC in carcinomas, adenomas, and non-neoplastic tissues were 6.30 ± 3.98, 0.78 ± 0.77 and 0.35 ± 0.33, respectively (Fig. 3 and Table 1). The average MCC in CRC was significantly higher (*p* < 0.05) than that in adenomas and non-neoplastic tissues. No significant difference was observed between MCC in adenomas and non-neoplastic tissues (*p* > 0.05). The MCs and TAMs were accentuated on the periphery of the neoplasm in the carcinomas and were observed in the supporting stroma in the adenomas. There was a weak positive correlation (*r* = 0.2; *p* > 0.05) between Ki-67 and MCC.

## Discussion

Ki-67 expression has been reported in association with malignancies in various human and animal tumors. Some studies [15] reported higher Ki-67 expression as an independent prognostic marker in human CRC, while others failed to demonstrate its prognostic significance [11]. TAMs [16–18] and MCs [25] are also involved in the development and progression of human colorectal tumors.

Human and dog CRCs are suggested to share similar molecular and genetic pathways of cancer development and progression [28]. Ki-67 expression and tumor associated inflammatory cells have not previously been documented in canine CRC. Here we report Ki-67 expression, TAMs and MCC in canine CRC. Ki-67 index was strongly (*r* = 0.71; *p* < 0.05) correlated with tumor malignancy. The results suggest association of Ki-67 with malignancy in canine CRC as was previously reported for human CRC [2, 15], prostate and breast cancers [14, 32] and various animal tumors [30, 33–35]. Because, there are genetic and pathogenic similarities between canine CRC and the human disease [36], in which Ki-67 prognostic value remained controversial [1, 14], its prognostic value in canine CRC is unknown at this time. Strong correlation between Ki-67 and mitotic indexes as observed in this study was expected since both parameters indicate degree of cell proliferation. We suggest that Ki-67 should be considered for a potential use to predict prognosis coupled with other measures of malignancy and should not be used as an independent prognostic marker in canine CRC. More studies should be done to determine its value as an independent prognostic marker for canine CRC.

Higher TAMs were documented in the colorectal adenomas (21.30 ± 20.70) and carcinomas (11.00 ± 9.82) than non-neoplastic tissues (7.69 ± 7.26) in this study. The TAMs count, however, was not directly associated with malignancy as a higher count was documented in adenomas than in carcinomas. TAMs count in CRC was positively correlated with mitotic (*r* = 0.35) and Ki-67 (*r* = 0.25) indices although the correlations were not statistically significant (*p* > 0.05). The higher TAMs count in adenomas and carcinomas than non-tumorous tissues suggests that TAMs may play a role in initiating and maintaining canine colorectal tumors. TAMs, conditioned by the tumor microenvironment are reported to have impact on cancer development by facilitating matrix invasion, angiogenesis, and tumor cell motility [37]. Furthermore, macrophages are immunosuppressive, and prevent tumor cell attack by natural killer and T-cells during tumor progression [38]. Because canine colorectal adenomas may progress to malignancy [39], most of malignant CRC arise from benign adenomatous polyps as observed in mouse model of CRC [36] and due to higher TAMs count in adenomas, we speculate that targeting TAMs would minimize development of adenomas, which would later progress to carcinomas. Similarly, depletion of pulmonary macrophages is suggested to be a strategy for attenuating lung cancer progression in humans [40]. Detailed study with large numbers of cases should be done to determine whether an early control of TAMs would help to control the development of canine colorectal tumors.

MCC was significantly higher in CRC than adenomas and non-neoplastic tissues and was strongly correlated with Ki-67 index indicating association of MCs with malignancy as was previously reported for various human [19, 20] and animal [21–23] neoplasms. Several published studies suggest that high MCC in CRC may play a role as an unfavorable prognostic marker [41]. Increased accumulation of mast cells within tumor environments has been correlated with poor prognosis, increased metastasis and reduced survival in several types of human cancer [20, 24]. Since benign colorectal tumors are characterized by high MCC [25] and showed significant remission upon MC depletion in mice, it was suggested that MC deserve consideration as a therapeutic target in polyposis and colon cancer [26].

## Conclusions

In summary, Ki-67 index, tumor associated macrophages and mast cells in canine CRC were recorded for the first time in this study. Ki-67 index, and MCC coupled with mitotic index and other clinical parameters may have a potential use in determining malignancy in canine CRC. Higher TAMs recorded in colorectal tumors than non-neoplastic tissues indicate that TAMs may play a role in the development of canine colorectal tumors. Targeting tumor associated inflammatory cells (TAMs and MCs) may be helpful in devising strategy to manage and minimize development of canine colorectal tumors. The data in this study provide a ground for further investigation of the clinical significance of the findings for chemopreventive and chemotherapeutic purposes in canine CRC.

## Abbreviations

CRC: Colorectal cancer
HIER: Heat Induced Epitope Retrieval
HPF: High power field
IHC: Immunohistochemistry
MC: Mast cell
MCC: Mast cell count
SD: Standard deviation
TAM: Tumor associated macrophages
